# Concurrent HSV1 Encephalitis and Carcinomatous Meningitis in a Stage IV Non-Small Cell Lung Cancer Patient: A Case Report

**DOI:** 10.51894/001c.143466

**Published:** 2025-09-30

**Authors:** Ho Young Curiel-Moon, Erica Wang, Isaac Turner

**Affiliations:** 1 College of Osteopathic Medicine Michigan State University; 2 Henry Ford Macomb, Michigan

## 74

### INTRODUCTION

Herpes Simplex Encephalitis (HSE) in itself is a rare but severe condition with a poor outcome, associated with a high mortality rate. Even with treatment with antiviral medication, many patients do not return to baseline function. Though sporadic cases of carcinomatous meningitis (CM) are exceedingly rare, CM can be a complication secondary to malignancy with aggressive disease progression and a grim prognosis of weeks to months. We report an unfortunate case of a cancer patient with concurrent infection of HSE and CM.

### CASE DESCRIPTION

A 50-year-old male patient with a history of metastatic non-small cell lung cancer was transferred to our care with complaints of fever and altered mental status. In the ICU, our patient presented with a vesicular rash, initially diagnosed as Herpes Zoster and a 14-day course of IV acyclovir. However, lumbar puncture and CSF serology revealed positive HSV1 PCR and EBV IgG antibody. Additionally, CSF cytology revealed CM. CM is often treated multimodally with a combination of surgery, intrathecal and systemic chemotherapy, and radiotherapy. At the time, intrathecal methotrexate was considered to treat CM but withheld due to fears of worsening HSE with immunosuppression. After education and discussions between the interprofessional team and the patient’s Durable Power of Attorney for Health Care (DPOA), the DPOA elected to complete the IV acyclovir course and discharge to hospice. The patient reached the end of his life four days after discharge.

### DISCUSSION/CONCLUSIONS

Here, we review an unusual case and progression of co-infection of HSV1 encephalitis and carcinomatous meningitis secondary to lung cancer, emphasizing the increased risk of overlapping infections in immunocompromised populations. This case underscores the challenges of treatment decisions; clinicians must carefully balance the urgency of antiviral therapy with the management of CM. Future studies in similar populations could identify and outline potential treatment options, concurrent or not.

